# Cavitary Lung Metastases in Prostate Cancer

**DOI:** 10.5334/jbsr.3008

**Published:** 2022-12-29

**Authors:** Felix Delbare, Geert Villeirs

**Affiliations:** 1 UZ Ghent, BE

**Keywords:** prostate cancer, excavated lung lesions, lung metastasis, CT-thorax, F18-PSMA-PET-CT

## Abstract

**Teaching Point:** Adenocarcinomas very rarely cause cavitary lung metastases.

## Case History

A 75-year-old man was diagnosed with prostate cancer in January 2019 (PSA 4.81 ng/mL, Gleason 3+4, pT3bN0M0). He was treated in June 2019 with robot-assisted laparoscopic-prostatectomy and pelvic lymph node dissection. Four months later, he received salvage intensity-modulated-radiotherapy to the prostatectomy bed for persistent PSA levels (0.23 ng/ml), in the absence of distant metastases on F18-PSMA-PET-CT. In February 2020, four months after local treatment, two mildly PSMA-avid para-aortic lymph nodes were disclosed on follow-up F18-PSMA-PET-CT and PSA-levels increased to 0.51 ng/ml. PSA was further monitored at regular intervals, but elevation to 0.74 ng/mL four months later prompted elective radiotherapy in June 2020.

**Figure 1:**
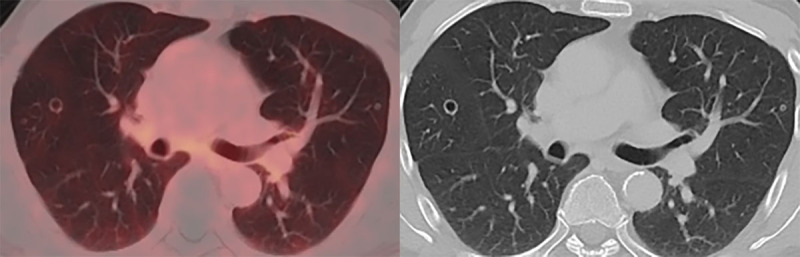


**Figure 2:**
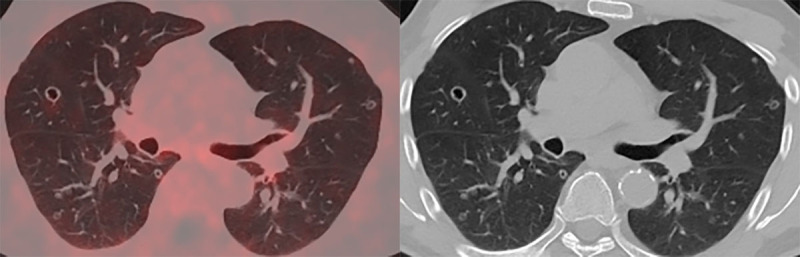


**Figure 3:**
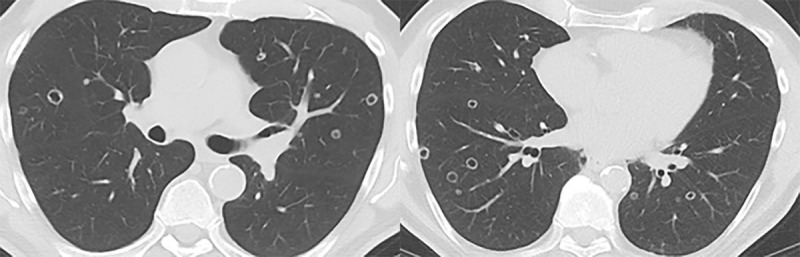


In November 2020, PSA-levels increased to 6 ng/mL and a third follow-up F18-PSMA-PET-CT revealed multiple, thin- and thick-walled, excavated, pericentrimetric, randomly distributed, nodular lung lesions with a slightly metabolic avid wall bilaterally (Figure 1) and enlarged para-iliac lymph nodes. The lymph nodes were treated with three sessions of stereotactic body radiation therapy in January 2021. The lung nodules were not treated, as their metastatic potential was considered low based on their morphology. A fourth F18-PSMA-PET-CT in May 2021 (Figure 2) and a CT-thorax in June 2021 (Figure 3) showed PSMA-uptake and increased volume and number of the cavitary lung nodules. A broad differential diagnosis of vasculitis, granulomatous disease, infectious/septic embolisms and atypical metastases was suggested.

Additional laboratory tests and bronchoscopy were not diagnostically conclusive, so a diagnostic pulmonary wedge resection was performed. The specimen contained two pericentrimetric lesions, both mildly PSA-positive and strongly NKX3.1-positive. NKX3.1 is a highly sensitive and specific tissue marker of metastatic prostatic adenocarcinoma. The diagnosis of pulmonary metastatic prostate cancer was made.

## Comments

Cavitary lung lesions are rarely metastatic and most commonly the primary site is a squamous cell carcinoma. Adenocarcinomas, such as prostate cancer, rarely present with cavitary metastases. Furthermore, there were no bone metastases which are the most frequent location of metastases in prostate cancer.

We only found two case reports of cavitary lung metastases in prostate cancer, both with a similar diagnostic pathway and only local therapy, though prostate cancer is one of the most frequent malignancies occurring in men. The prevalence therefore is extremely low, but there are important implications. The therapeutic options differ for nonmetastatic, versus oligo- or polymetastatic (>3) disease. Polymetastatic disease requires a systemic approach, whereas in all other cases patients may still benefit from local radiotherapy/surgery.

The diagnosis of cavitary prostatic lung metastases cannot be made through imaging alone, although the radiologist should be aware that cavitary lung lesions may be related to prostate cancer.

Several pathological processes can result in cavitation including different types of necrosis, cystic dilatation of lung structures, internal cyst formation or internal desquamation of tumour cells with subsequent liquefaction. Moreover, the likelihood of occurrence of cavitation depends on host factors and the nature of the pathogenic process [1]. None fit our patient entirely, so the aetiology remains uncertain.
